# Supplementary material for: Implementation of KEIGAAF in Primary Schools: A Mutual Adaptation Physical Activity and Nutrition Intervention
Source: Int J Environ Res Public Health. 2020 Jan 24;17(3):751. doi: 10.3390/ijerph17030751 (PMC7037055; doi:10.3390/ijerph17030751)
Supplement: Supplementary file 1 [file ijerph-17-00751-s001.zip › Manuscript Verjans-Janssen et al (IJERPH).revised_Supplemental file 1.docx]

**Supplemental file 1. Interview Guide KEIGAAF**

**Theoretical Framework**

Consolidated Framework for Implementation Research (Damschroder et al., 2009)

**Basic questions working group chair/school principal:**

1. What is KEIGAAF according to you?
2. What is your role in the KEIGAAF approach? / How are you involved in the KEIGAAF approach?
3. What is the most important reason for you to participate in KEIGAAF?
4. What is the aim of the KEIGAAF approach, according to you?
5. What is your opinion on the KEIGAAF approach?

----------------

1. How is the KEIGAAF approach organized within your school?
2. Was there a moment in time that the KEIGAAF approach was becoming successful? If so, can you describe this moment?
3. Was there a moment in time that the KEIGAAF approach was not so successful? If so, can you describe this moment?
4. Which difficulties do you experience with implementing the KEIGAAF approach?
5. What facilitates implementation of the KEIGAAF approach?

----------------

1. How does a succesfull KEIGAAF approach look like, according to you? Does your approach meet that description? Why/why not?
2. Who or what is crucial for school health promotion at your school? Are these factors present?
3. What would you like to advise schools who are willing to implement the KEIGAAF approach?

----------------

1. Will the KEIGAAF approach be continued at your school? If so, how and in which form? And what will your role be in this continuation?
2. What is necessary to continue the KEIGAAF approach?

**Basic questions member of steering committee:**

1. What is KEIGAAF according to you?
2. What is your role/the role of your organization in the KEIGAAF approach? / How are you/is your organization involved in the KEIGAAF approach?
3. What is the most important reason for you to participate in KEIGAAF?
4. What is the aim of the KEIGAAF approach, according to you?
5. What is your opinion on the KEIGAAF approach?

----------------

1. What is the function of the steering committee in the KEIGAAF approach?
2. Could you describe the collaboration with the other members of the steering committee in the KEIGAAF approach?
3. Do you have sight at the implementation of the KEIGAAF approach at the schools? If so, how (successful) is the KEIGAAF approach implemented at school? / (What influences this successful/unsuccessful implementation?)
4. Was there a moment in time that the KEIGAAF approach was becoming successful? If so, can you describe this moment?
5. Was there a moment in time that the KEIGAAF approach was not so successful? If so, can you describe this moment?
6. Which difficulties do you experience with guiding the KEIGAAF approach?
7. What facilitates guidance of the KEIGAAF approach?

----------------

1. How does a successful KEIGAAF approach look like, according to you? Does the KEIGAAF approach at the schools meet that description? Why/why not?
2. Who or what is crucial for school health promotion? Are these factors present?
3. What would you like to advise schools who are willing to implement the KEIGAAF approach?

----------------

1. Will the KEIGAAF approach be continued? If so, how and in which form? And what will your role be in this continuation?
2. What is necessary to continue the KEIGAAF approach?
