# Supplementary material for: Implementation of KEIGAAF in Primary Schools: A Mutual Adaptation Physical Activity and Nutrition Intervention
Source: Int J Environ Res Public Health. 2020 Jan 24;17(3):751. doi: 10.3390/ijerph17030751 (PMC7037055; doi:10.3390/ijerph17030751)
Supplement: Supplementary file 1 [file ijerph-17-00751-s001.zip › Manuscript Verjans-Janssen et al (IJERPH).revised_Supplemental file 2.docx]

**Table A. Schools’ physical activity promotion at the end of the intervention period.**

|  | **School 1** | **School 2** | **School 3** | **School 4** |
| --- | --- | --- | --- | --- |
| **Physical education** | | | | |
|  | 60 minutes PE provided by PE-teacher | 2x 45 minutes PE of which 45 minutes PE provided by PE-teacher and the other 45 minutes by schoolteacher  Sports clinics during PE | 2x 45 minutes PE of which 45 minutes PE provided by PE-teacher and the other 45 minutes by schoolteacher  *Sports clinics during PE* | 60 minutes PE provided by PE-teacher and 45 minutes outdoors PE provided by schoolteacher  *Sports clinics during PE* |
| **Physical activity during school** | | | | |
| **During recess** | *PA activities during recess three times/week*  PA material during both breaks | *PA activities during recess one time/week*  *PA material during both breaks* | *PA activities during recess one time/week*  PA material during both breaks | *PA activities during recess one time/week*  *PA material during both breaks* |
| **During educational hours** | *Active curriculum*  *Activity breaks* | *Active curriculum*  *Activity breaks*  *Energizers* | *Activity breaks* | *Activity breaks*  *Active curriculum* |
| **Occasional activities** | *Sports day* | Sports day  Sports week  National outside play day | Sports day  Sports week | *PA and healthy nutrition week*  Sports day  Sports week  *Daily Mile* |
| **PA before and after school** | | | | |
| **Continuous activities** | *Afterschool PA monthly*  *Active transport activity once a month* | Afterschool PA weekly | Afterschool PA monthly  Active transport activity once a month | *Afterschool PA monthly* |
| **Occasional activities** | *Active transport-week* | Local ice-skating event  Local marathon | Active transport-week  Local marathon  Local ice-skating event | Local marathon  Local ice-skating event |
| **Staff involvement in school PA policies/activities** | | | | |
|  | *High degree of stimulation of PA activities during recess.*  *High degree of active transport stimulation.*  High degree of implementation of school PA policies. | *Moderate degree of stimulation of PA activities during recess.*  *Moderate degree of active transport stimulation.*  Moderate degree of implementation of school PA policies. | *High degree of stimulation of PA activities during recess.*  High degree of active transport stimulation.  High degree of implementation of school PA policies. | *High degree of stimulation of PA activities during recess.*  Very high degree of active transport stimulation.  Moderate degree of implementation of school PA policies. |
| **Parental engagement in school PA activities** | | | | |
|  | Activities during recess, implementing sports days and active transport stimulation. | Sports days. | Activities during recess, implementing sports days and active transport stimulation. | Sports days and active transport stimulation. |
|  | | | | |
|  | **School 5** | **School 6** | **School 7** | **School 8** |
| **Physical education** | | | | |
|  | 2x 45 minutes PE of which 45 minutes PE provided by PE-teacher and the other 45 minutes by schoolteacher | 50 minutes PE provided by PE-teacher and 45 minutes outdoors PE provided by schoolteacher  *Sports clinics during PE* | 2x 45 minutes PE of which 45 minutes PE provided by PE-teacher and the other 45 minutes by schoolteacher  Sports clinics during PE | 2x 45 minutes PE of which 45 minutes PE provided by PE-teacher and the other 45 minutes by schoolteacher  Sports clinics during PE |
| **Physical activity during school** | | | | |
| **During recess** | *PA material during both breaks* | *PA activities during recess one time/week*  *PA material during long break* | PA activities during recess four times/week  *PA material during both breaks* | PA activities during recess one time/week  *PA material during both breaks* |
| **During educational hours** | Activity breaks | *Active curriculum*  *Standing learning*  *Activity breaks* | *Active curriculum*  *Standing desks / Standing learning* | Active curriculum |
| **Occasional activities** | Sports day | Sports day | Sports day  Sports week | Sports day |
| **PA before and after school** | | | | |
| **Continuous activities** |  | After school PA monthly | After school PA weekly | After school PA monthly |
| **Occasional activities** | Local marathon | Local marathon  Local ice-skating event  School soccer tournament  Active transport-week | Local marathon  *Three day walk through neighborhood* | Local marathon  Local ice-skating event  Three day walk through neighborhood |
| **Staff involvement in school PA policies/activities** | | | | |
|  | Moderate degree of stimulation of PA activities during recess.  High degree of active transport stimulation.  Moderate degree of implementation of school PA policies. | *Moderate degree of stimulation of PA activities during recess.*  Moderate degree of active transport stimulation.  N/A (no school PA policy) | High degree of stimulation of PA activities during recess.  Moderate degree of active transport stimulation.  High degree of implementation of school PA policies. | Low degree of stimulation of PA activities during recess.  Low degree of active transport stimulation.  Moderate degree of implementation of school PA policies. |
| **Parental engagement in school PA activities** | | | | |
|  | Sports days. | Afterschool activities and sports days. | Sports days. | Sports days. |

*Note:* activities and actions in italics were implemented or enhanced during the intervention period (activities and actions in normal font were already present at the start of the intervention).

**Table B. Schools’ healthy nutrition promotion at the end of the intervention period.**

|  | **School 1** | **School 2** | **School 3** | **School 4** |
| --- | --- | --- | --- | --- |
| **Nutrition education** | | | | |
|  | *Nutrition education: educational program “Tasty and Healthy”, lessons of EU-schoolfruit* | Nutrition education: lessons of “I eat it better” and EU-schoolfruit | *Nutrition education: educational program “Taste Lessons”, lessons of “I eat it better”* | *Nutrition education: educational program “Taste Lessons”, lessons of “I eat it better”* |
| **Healthy nutrition during school** | | | | |
| **Continuous activities** | School fruit  *Water bottles* | School fruit  Water bottles | School fruit  *Water bottles* | School fruit |
| **Occasional activities** | *Healthy breakfast week* Breakfast activities  *Healthy nutrition week* | Breakfast activities  Healthy snack week | Breakfast activity  Healthy snack week | *Breakfast activities*  *Healthy snack week*  *Water promotion week*  *PA and healthy nutrition week* |
| **Nutrition policy** | *Written nutrition policy concerning healthy snacks, lunch, drinks, birthday treats, and modeling healthy nutrition behavior by teachers.* | *Written nutrition policy concerning healthy snacks, lunch, drinks and modeling healthy nutrition behavior by teachers.* | *Written nutrition policy concerning healthy snacks, lunch, drinks, birthday treats, and modeling healthy nutrition behavior by teachers.* | *Written nutrition policy concerning healthy snacks, lunch, drinks, birthday treats, and modeling healthy nutrition behavior by teachers.* |
| **Healthy nutrition before and after school** | | | | |
|  | Cooking workshop |  |  | Healthy nutrition and birthday treats at out-of-school care center |
| **Staff involvement in school nutrition policy/activities** | | | | |
|  | *High degree of implementation of school nutrition policies.*  More than half of staff members support the school nutrition policies. | *Moderate degree of implementation of school nutrition policies.*  All staff members support the school nutrition policies. | *High degree of implementation of school nutrition policies.*  All staff members support the school nutrition policies. | *High degree of implementation of school nutrition policies.*  All staff members support the school nutrition policies. |
| **Parental engagement in school nutrition activities** | | | | |
|  | *Parents involved in school nutrition education, nutrition policy and nutrition activities.* | *Parents involved in school nutrition policy.* |  | *Parents involved in school nutrition education, nutrition policy and nutrition activities.* |
|  |  |  |  |  |
|  | **School 5** | **School 6** | **School 7** | **School 8** |
| **Nutrition education** |  |  |  |  |
|  | *Educational program “Tasty and Healthy”,* lessons of EU-schoolfruit | *Educational program “Taste Lessons”, lessons of “I eat it better”, EU-schoolfruit and national Breakfast event* | Lessons of EU-school fruit and cooking workshops | *Educational program “Tasty and Healthy”,* lessons of EU-schoolfruit |
| **Healthy nutrition during school** | | | | |
| **Continuous activities** | School fruit  Vegetable garden | School fruit  *Water jugs* | School fruit  Vegetable garden | School fruit  *Water bottles* |
| **Occasional activities** | Breakfast activities  Tap water day | Breakfast activities  Tap water day  *Healthy snack week*  *Fruit action month*  *Healthy birthday treats session* |  | Breakfast activities |
| **Nutrition policy** | Unwritten nutrition policy concerning lunch and drinks | *Written nutrition policy concerning healthy snacks, lunch, drinks, birthday treats, and modeling healthy nutrition behavior by teachers* | Written nutrition policy concerning healthy snacks, lunch, drinks and birthday treats | *Written nutrition policy concerning healthy snacks, drinks, birthday treats, and modeling healthy nutrition behavior by teachers* |
| **Healthy nutrition before and after school** | | | | |
|  | *Water tap at nearby playground* |  |  |  |
| **Staff involvement in school nutrition policy/activities** | | | | |
|  | Moderate degree of implementation of school nutrition policies. | *High degree of implementation of school nutrition policies.* | High degree of implementation of school nutrition policies. | *High degree of implementation of school nutrition policies.* |
| **Parental engagement in school nutrition activities** | | | | |
|  | *School nutrition education* | *School nutrition policy* | *School nutrition activities* | *School nutrition policy* |

*Note:* activities and actions in italics were implemented or enhanced during the intervention period (activities and actions in normal font were already present at the start of the intervention).
